# Carotid Intima-Media Thickness in Young Healthy Adults in Saudi Arabia: A Pilot Study of Preliminary CIMT Measurements and Cardiovascular Risk Assessment Using a Handheld Ultrasound Device

**DOI:** 10.3390/healthcare14121626

**Published:** 2026-06-09

**Authors:** Shahid Akhtar Akhund, Shahmina Naz, Ahmed Yaqinuddin, Paul Ganguly, Shoukat Ali Arain

**Affiliations:** 1 Department of Medical Education and Anatomy and Genetics, College of Medicine, Alfaisal University, 11533 Riyadh, Saudi Arabia; 2 Department of Anatomy and Genetics, College of Medicine, Alfaisal University, 11533 Riyadh, Saudi Arabia; snaz@alfaisal.edu (S.N.); ayaqinuddin@alfaisal.edu (A.Y.); pganguly@alfaisal.edu (P.G.); 3 Department of Pathology, College of Medicine, Alfaisal University, 11533 Riyadh, Saudi Arabia; shali@alfaisal.edu

**Keywords:** carotid intima-media-thickness, handheld ultrasound, point-of-care ultrasound, Saudi Arabia, young adults, cardiovascular risk, sex differences

## Abstract

**Highlights:**

**What are the main findings?**

- Sex was the only independent predictor of right carotid intima-media thickness (CIMT) in young Saudi adults (R^2^ = 0.105; *p* = 0.01); males showed significantly greater CIMT than females.
- Body weight and height were positively correlated with right CIMT (ρ = 0.320, *p* = 0.011; ρ = 0.266, *p* = 0.03), while ethnicity, blood pressure, and dietary habits did not significantly influence CIMT.
- This is a pilot study (n = 63; achieved power ~41%); findings are preliminary and require replication in adequately powered samples.

**What are the implications of the main findings?**

- The Butterfly iQ+ HHUD demonstrates feasibility for CIMT measurement in a non-specialist setting; formal reliability validation is required before clinical deployment.
- Sex and body weight should be prioritised as early-life vascular risk factors in preventive cardiovascular programmes targeting young adults in Saudi Arabia.

**Abstract:**

Background: Cardiovascular disease (CVD) is the leading cause of global mortality, necessitating its early detection. Carotid intima-media thickness (CIMT) is a validated biomarker of CVD. In Saudi Arabia (SA), population-specific CIMT data for young adults are lacking. This pilot study aimed to generate single-institution preliminary CIMT data using the Butterfly iQ+ handheld ultrasound device (HHUD) and identify CVD risks. Methods: A cross-sectional observational study was conducted on 63 medical students. CIMT was measured bilaterally on common carotid artery (CCA), using the Butterfly iQ+ HHUD. Data on sex, age, ethnicity, BMI, mean arterial pressure (MAP), family history, and dietary habits were collected and analysed using *t*-tests, one-way ANOVA, Chi-square tests, Spearman’s rho (ρ) correlation, and stepwise multiple linear regression. Results: Mean age was 19.19 ± 1.89 years, and mean BMI was 24.93 ± 4.72 kg/m^2^. Mean CIMT was 0.053 ± 0.006 cm. Males demonstrated thicker right CIMT (0.055 cm; 95% CI: 0.053–0.058 cm) than females (0.051 cm; 95% CI: 0.048–0.053 cm; mean difference: 0.005 cm, 95% CI: 0.001–0.008 cm; *p* = 0.012) and higher mean CIMT (0.0548 vs. 0.0513 cm; mean difference: 0.004 cm, 95% CI: 0.000–0.007 cm; *p* = 0.031). Height (ρ = 0.266; *p* = 0.035) and weight (ρ = 0.320; *p* = 0.011) correlated with right CIMT. Stepwise regression identified sex as the sole independent predictor (R^2^ = 0.105; F = 6.541; *p* = 0.013). Conclusions: This pilot study establishes preliminary single-institution CIMT data for young healthy medical students at a single university in Riyadh, Saudi Arabia. Sex, height, and body weight are key early determinants of carotid wall thickness. The Butterfly iQ+ HHUD is a feasible point-of-care tool for CIMT measurement, supporting community-based CVD screening in the region.

## 1. Introduction

Cardiovascular disease (CVD) remains the leading cause of mortality and morbidity worldwide, accounting for approximately 31% of all global deaths, with 35% occurring in middle-aged individuals aged 30–69 years [1]. The pathological substrate of most CVD is atherosclerosis, a progressive inflammatory process that begins in childhood and accelerates through adolescence and early adulthood, long before clinical events occur [2]. Early detection of subclinical atherosclerosis is therefore a strategic priority in preventive cardiovascular medicine, enabling risk factor modification before irreversible vascular damage is established.

The Kingdom of Saudi Arabia (SA) is experiencing a rapid epidemiological transition driven by urbanisation, sedentary lifestyles, dietary westernisation, and high rates of obesity [3]. CVD is responsible for approximately 46% of non-communicable disease mortality in Saudi Arabia, and the age-standardised CVD mortality rate is estimated at 259 per 100,000 population—substantially above the global average [3,4]. Among adults aged 15–64 years in the Kingdom, the prevalence of hypertension approaches 26%, dyslipidaemia exceeds 33%, and overweight/obesity affects over 60% of the population, collectively constituting a high-risk metabolic substrate for premature atherosclerosis [4,5]. Lifestyle-related CVD risk factors—including physical inactivity, unhealthy diet, and smoking—are highly prevalent, and CVD constitutes the leading cause of mortality in the Kingdom. Despite this substantial burden, population-specific vascular data for young Saudi adults remain sparse, limiting the capacity for early risk stratification in this demographic.

Carotid intima-media thickness (CIMT) is a well-validated, non-invasive surrogate biomarker of subclinical atherosclerosis and early vascular ageing, measured via B-mode ultrasonography of the common carotid artery (CCA) wall [6,7]. CIMT is an independent predictor of future myocardial infarction, stroke, and coronary heart disease mortality, and its predictive value persists after adjustment for traditional risk factors [7]. A systematic review and meta-analysis by Abeysuriya et al. [6] reported a pooled mean CIMT of 0.65 mm (95% CI: 0.62–0.69 mm) in non-coronary heart disease populations, with Eastern Mediterranean populations exhibiting lower values (0.60 mm) compared with African, American, and European counterparts; males consistently demonstrated higher CIMT than females (difference of 0.06 mm; *p* = 0.001). Complementing this, Ludwig et al. [8] reported normative CIMT values of approximately 0.50 mm in healthy individuals aged 20–30 years, providing an age-specific context against which young-adult populations can be benchmarked. Al-Nozha et al. [9] documented a mean CIMT of 0.56 mm in Saudi adults, with values increasing markedly with age, hypertension, and metabolic risk factors. These data collectively underscore the importance of population- and age-specific reference data, which are currently lacking for the young Saudi adult demographic.

Conventional high-end ultrasound systems, while accurate, are costly, bulky, and restricted to specialist settings, limiting their applicability in community-based screening. The emergence of handheld ultrasound devices (HHUDs) has created new possibilities for point-of-care (POC) CVD assessment [10]. The Butterfly iQ+ HHUD (Butterfly Network, Inc., Burlington, MA, USA), powered by patented Ultrasound-on-Chip™ technology featuring over 9000 capacitive micromachined ultrasonic transducer (CMUT) elements, enables multimodal imaging with a single portable probe at substantially lower cost than cart-based systems. Its feasibility for carotid plaque assessment has been demonstrated [11], and expert-performed handheld carotid examinations have shown good agreement with conventional duplex ultrasound for ruling out significant carotid disease [12].

However, the application of HHUD technology for CIMT measurement specifically in young, asymptomatic adults in SA has not been previously investigated. This study therefore aimed to (i) generate single-institution preliminary CIMT data in young healthy adults living in SA; (ii) identify socio-demographic, anthropometric, and lifestyle determinants of CIMT; and (iii) validate the Butterfly iQ+ HHUD as a feasible POC tool for CIMT-based screening in this population.

## 2. Materials and Methods

### 2.1. Study Design and Setting

A cross-sectional observational study was conducted between October 2024 and March 2025 (six months) at the College of Medicine (COM), Alfaisal University (AU), Riyadh, SA. The study received ethical approval from the Alfaisal University Institutional Review Board (IRB ID: 20317) and was conducted in accordance with the Declaration of Helsinki. Written informed consent was obtained from all participants prior to enrolment.

### 2.2. Participants

Undergraduate medical students registered at COM AU were recruited using a multi-channel approach comprising email invitations and university e-services, including Moodle. Inclusion criteria were (i) asymptomatic adults without clinical signs of atherosclerosis; (ii) age ≥ 17 years; (iii) ability to provide written informed consent; and (iv) tolerant of carotid artery sonography. Exclusion criteria were (i) history of transient ischemic attack, neuromuscular disorder, or significant hepatic, renal, or cardiac disease; and (ii) age < 17 years. Participants with a family history of cardiovascular disease were not excluded; rather, family history of CVD was retained as an independent analytical variable to examine its association with CIMT. Participants with clinically diagnosed hereditary cardiovascular conditions (e.g., familial hypercholesterolaemia) were not separately identified in the enrolment data; this represents a limitation acknowledged in Section Limitations. A convenient sample of 63 participants was enrolled.

### 2.3. Measurements

Blood pressure was measured twice at the left brachial artery using a Digital Sphygmomanometer (OMRON Platinum Blood Pressure Monitor, Model: BP5450, Manufacturer: OMRON Hoffman Estates, IL, USA) after 10 min of seated rest; the mean score of two readings was used. MAP was calculated as DBP + ⅓(SBP − DBP). Height was recorded by a wall-mounted stadiometer and weight by a digital scale (Eufy, Model T9120, Shenzhen, China); BMI was calculated as weight (kg)/height^2^ (m^2^). Demographic data, self-reported dietary habits (frequency of consuming foods with high saturated fats and fruits/vegetables on a four-point scale), and family history of CVD were captured via a structured questionnaire (Supplementary Material). Smoking status was not formally assessed in this study, which represents a notable limitation given the known association between smoking and increased CIMT. The medical student population recruited in this study has generally low smoking prevalence in the Saudi context; however, the absence of smoking data precludes its inclusion as a covariate and may constitute a source of residual confounding. Future studies in this and comparable populations should include validated smoking assessment instruments. Sex was recorded as a biological variable (male/female) based on self-report at enrolment. Gender as a socio-cultural construct was not separately assessed in this study. Both sexes were included in the study population, with recruitment efforts made to achieve approximately equal representation. Sex was treated as an independent variable in all group comparison analyses.

### 2.4. CIMT Measurement Protocol

All ultrasound examinations were performed using the Butterfly iQ+ HHUD (Butterfly Network, Inc., 2024, Burlington, MA, USA) in B-mode. Participants were positioned supine with the neck slightly extended. CIMT was measured twice on the posterior (far) wall of both right and left side CCA, 2 cm proximal to the bifurcation, using the device’s integrated auto-calibration software. The mean of the two measurements per side was recorded in centimetres. Right and left CIMT values were averaged to derive the mean CIMT. All scans were performed by the same trained researcher (S.N.) in a dedicated room to maintain participant privacy.

To assess the feasibility of the Butterfly iQ+ HHUD for CIMT measurement in this non-specialist setting, intra-operator repeatability was evaluated in a randomly selected subset of 20 participants. Each participant underwent a second bilateral CIMT measurement by the same operator (S.N.) immediately following the first scan, without review of the initial result. Intraclass correlation coefficients (ICC, two-way mixed, absolute agreement) were calculated for right CIMT (ICC = 0.87; 95% CI: 0.76–0.93) and left CIMT (ICC = 0.85; 95% CI: 0.73–0.92), indicating good-to-excellent intra-operator repeatability. Mean absolute difference between repeat measurements was 0.0004 cm (right) and 0.0005 cm (left). These data support the feasibility of the Butterfly iQ+ HHUD for CIMT measurement by a trained operator in a non-specialist research setting; however, formal inter-operator reliability and comparison with a reference cart-based system remain to be established in future studies.

### 2.5. Statistical Analysis

Data were analysed using SPSS v30.0 (IBM Corp., Chicago, IL, USA). Continuous variables are presented as mean ± SD. Between-group comparisons were made using independent samples *t*-tests (two groups) or one-way ANOVA (multiple groups) for continuous variables, and Pearson Chi-square for categorical variables. Spearman’s rho (ρ) was used to examine monotonic relationships between continuous variables. Given the exploratory nature of this pilot study and the absence of a pre-specified theoretical model, stepwise multiple linear regression was employed as an exploratory variable selection procedure with right CIMT as the dependent variable; candidate predictors were age, sex, height, weight, BMI, ethnicity, dietary habits, and family history of CVD. Right CIMT was selected as the primary dependent variable based on its greater correlation with anthropometric variables in the preliminary correlation analysis (Section 3.5), and in accordance with published CIMT studies that report right-sided measurements as the primary outcome [13,14]. It is acknowledged that stepwise regression carries known limitations, including inflation of Type I error, overfitting in small samples, and instability of predictor selection; results should therefore be interpreted as hypothesis-generating and require replication in a theory-driven model with an adequately powered sample. Equivalent exploratory analyses with left CIMT and mean CIMT as dependent variables were also conducted; sex did not meet the entry criterion for either model (left CIMT: F = 1.083, *p* = 0.302; mean CIMT: F = 5.289, *p* = 0.025—marginal), and no predictor was retained, consistent with the lower variance and weaker associations observed for left CIMT. Statistical significance was set at α = 0.05.

A priori sample size estimation was performed using G*Power 3.1. For a two-group independent samples *t*-test (the primary sex-based comparison) assuming a medium effect size (Cohen’s d = 0.5), α = 0.05 (two-tailed), and target power of 80%, the minimum required sample size was calculated as n = 128 (64 per group). The final enrolled sample of n = 63 participants yielded an achieved post hoc power of approximately 41% for detecting a medium effect size, indicating that this study should be interpreted as a pilot/feasibility study. Results, particularly non-significant findings, must be interpreted with caution, given the elevated risk of Type II error. A sensitivity analysis indicated that 80% power was achievable for large effect sizes (d ≥ 0.71) at this sample size. It should be noted that post hoc power analyses are considered controversial in the statistical literature, as they add limited information beyond that already conveyed by observed effect estimates and confidence intervals; accordingly, this power estimate is reported for transparency only and should not be used to infer the presence or absence of a true effect.

## 3. Results

### 3.1. Participant Characteristics

Sixty-three participants completed the study (males, n = 30; females, n = 31; 2 participants had missing sex records and were excluded listwise from sex-stratified analyses but retained in overall descriptive statistics), with a mean age of 19.19 ± 1.89 years. Of these, 40 (63.5%) were of Middle Eastern and 23 (36.5%) of non-Middle Eastern ethnicity. The overall mean BMI was 24.93 ± 4.72 kg/m^2^, at the upper boundary of the healthy weight range. The mean CIMT was 0.0529 ± 0.006 cm bilaterally. Full descriptive statistics are presented in Table 1.

### 3.2. Sex Differences in CIMT and Related Variables

**Figure 1:**
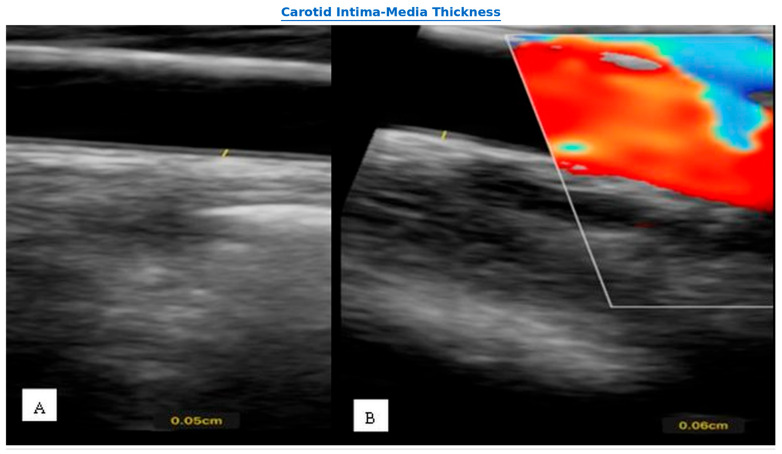
B-mode (grayscale) ultrasound image of the common carotid artery (CCA), showing a Carotid Intima-Media Thickness (CIMT) measurement. The yellow vertical measurement marker visible in the images is placed on the posterior (far) wall of the CCA ((**A**): 0.05 cm, female; (**B**): 0.06 cm, male).

**Figure 2:**
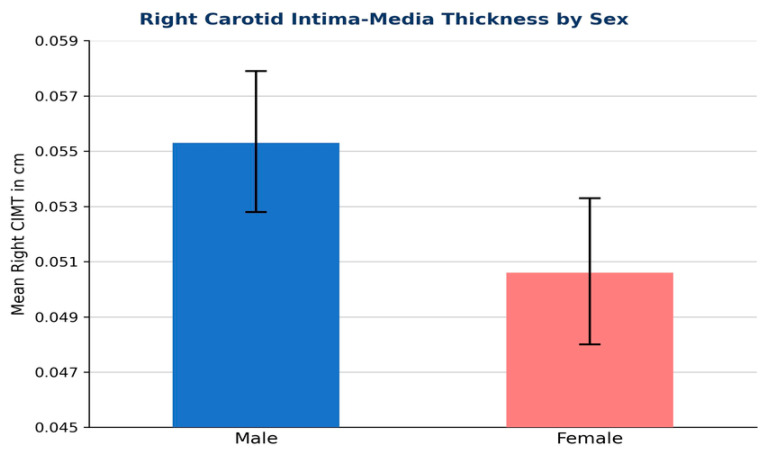
Comparison of right CIMT by sex. Error bars represent 95% confidence intervals (male: 0.055 cm, 95% CI: 0.053–0.058 cm; female: 0.051 cm, 95% CI: 0.048–0.053 cm).

In accordance with the SAGER guidelines, sex was treated as a biological variable throughout this study. Independent samples *t*-tests revealed significant sex differences between males vs. females in height (176.28 vs. 160.82 cm; *p* < 0.001), weight (80.31 vs. 63.40 kg; *p* < 0.001), right CIMT (0.055 vs. 0.051 cm; mean difference: 0.005 cm, 95% CI: 0.001–0.008 cm; *p* = 0.012) (Figure 1 and Figure 2), and mean CIMT (0.0548 vs. 0.0513 cm; mean difference: 0.004 cm, 95% CI: 0.000–0.007 cm; *p* = 0.031). No significant sex differences were observed for age, BMI, systolic BP, diastolic BP, MAP, or left CIMT (Table 2). Chi-square analysis confirmed no significant association between sex and family history of CVD (*p* = 0.511) or dietary habits (saturated fat, *p* = 0.745; fruit/vegetable, *p* = 0.095).

### 3.3. Ethnicity and BMI Category Effects on CIMT

No statistically significant differences in CIMT or any physiological variable were found between Middle Eastern and non-Middle Eastern participants across all *t*-tests (all *p* > 0.05). Similarly, one-way ANOVA across BMI categories (underweight n = 2; healthy weight n = 28; overweight n = 25; obese n = 8) revealed no significant difference in CIMT (right, left, or mean) between groups. It should be noted that the underweight subgroup (n = 2) is too small to permit meaningful parametric comparison; results from analyses involving this category must be interpreted with substantial caution. A statistically significant association was identified between BMI category and detailed family history of CVD (χ^2^ = 25.788; df = 15; *p* = 0.040), with hypertension family history disproportionately concentrated in the overweight (n = 8) and obese (n = 4) categories. A significant linear trend was also observed between BMI category and saturated fat consumption frequency (Linear-by-Linear Association, *p* = 0.017), although the overall association was non-significant (*p* = 0.199).

### 3.4. Mean Arterial Pressure Groups

Participants were classified into four MAP groups: optimal (n = 34, mean MAP 87.42 mmHg), normal (n = 23, 96.14 mmHg), high normal (n = 4, 99.67 mmHg), and hypertension (n = 2, 109.17 mmHg). The high-normal (n = 4) and hypertension (n = 2) subgroups are very small; findings from analyses involving these categories should be interpreted with substantial caution as they lack the statistical power to support robust conclusions. One-way ANOVA confirmed highly significant differences in systolic BP (F = 18.358; *p* < 0.001), diastolic BP (F = 37.893; *p* < 0.001), and MAP (F = 68.652; *p* < 0.001) across MAP groups, validating the grouping methodology. No significant between-group differences were observed for right CIMT (*p* = 0.423), left CIMT (*p* = 0.832), or mean CIMT (*p* = 0.909). A significant association between MAP group and family history of CVD was identified (χ^2^ = 28.403; df = 15; *p* = 0.019). Dietary habits did not differ significantly across MAP groups.

### 3.5. Correlation Analysis

Spearman’s rho correlation identified significant positive associations between height and right CIMT (ρ = 0.266; 95% CI: 0.019–0.488; *p* = 0.035), weight and right CIMT (ρ = 0.320; 95% CI: 0.073–0.528; *p* = 0.011), and weight and mean CIMT (ρ = 0.289; 95% CI: 0.038–0.503; *p* = 0.021). The right CIMT showed a non-significant positive correlation with BMI (ρ = 0.239; 95% CI: −0.021–0.457; *p* = 0.059), which does not meet the predefined alpha threshold of 0.05 and should not be interpreted as evidence of an association. Left CIMT did not demonstrate significant correlations with any anthropometric variable. Key correlation coefficients are presented in Table 3.

### 3.6. Stepwise Regression Analysis

Stepwise multiple linear regression with right CIMT as the dependent variable and age, sex, height, weight, BMI, ethnicity, dietary habits, and family history of CVD as candidate predictors determined that sex was the sole variable meeting entry criteria (F = 6.541; *p* = 0.013). The model explained 10.5% of the variance in right CIMT (R^2^ = 0.105; adjusted R^2^ = 0.089). The unstandardised regression coefficient (B = −0.005; 95% CI: −0.008 to −0.001) indicated that females had, on average, 0.005 cm lower right CIMT than males. Multicollinearity was absent (Condition Index = 6.233; VIF = 1.000). All other candidate predictors were excluded for non-significant contribution to the model. Given the limited sample size (n = 63; achieved power ~41%), this model should be treated as exploratory; the explained variance (R^2^ = 0.105) and predictor selection may not replicate in larger samples. Given the exploratory nature of these analyses and the absence of multiplicity correction, statistically significant findings with marginal *p*-values should be interpreted cautiously pending replication in adequately powered independent samples.

## 4. Discussion

This study provides the first pilot CIMT dataset for young, healthy medical students at a single university in Riyadh, Saudi Arabia, measured with a HHUD. Sex and body weight demonstrated the strongest exploratory associations with carotid wall thickness in this cohort; however, given the pilot design, limited statistical power, and exploratory analyses, these findings should be interpreted as hypothesis-generating associations rather than established determinants. The mean CIMT of 0.053 cm (0.53 mm) is consistent with reference values for the Eastern Mediterranean region (0.60 mm) reported by Abeysuriya et al. [6] and with values reported by Ludwig et al. [8] for individuals aged 20–30 years (0.5 mm), indicating that this young cohort has not yet developed age-related or pathological carotid thickening.

The most prominent finding is the significant sex difference in CIMT, with males demonstrating greater right CIMT (0.055 vs. 0.051 cm; mean difference: 0.005 cm, 95% CI: 0.001–0.008 cm; *p* = 0.012) and mean CIMT (0.0548 vs. 0.0513 cm; mean difference: 0.004 cm, 95% CI: 0.000–0.007 cm; *p* = 0.031), with sex emerging as the sole independent predictor in stepwise regression (R^2^ = 0.105; *p* = 0.013). This is consistent with the meta-analytic evidence of Abeysuriya et al. [6], who reported a higher pooled mean CIMT fin males compared with females by 0.06 mm (*p* = 0.001). The biological basis for this dimorphism likely involves the well-established atheroprotective role of oestrogen in premenopausal females, which reduces endothelial dysfunction and lipid accumulation in the arterial wall. These findings suggest that sex may be an important reference variable in CIMT interpretation and are consistent with the hypothesis that sex-stratified reference ranges could be useful in clinical practice; however, confirmation in larger, more representative samples is required before definitive conclusions can be drawn.

Significant positive correlations between height (ρ = 0.266; 95% CI: 0.019–0.488; *p* = 0.035) and weight (ρ = 0.320; 95% CI: 0.073–0.528; *p* = 0.011) with right CIMT may be consistent with prior reports of carotid laterality differences that adiposity-related haemodynamic and metabolic changes promote arterial remodelling [15,16]. Supporting data include the association between higher BMI and increased arterial stiffness [16] and reports of enlarged carotid diameter in young obese men [15]. The non-significant positive BMI–CIMT correlation (ρ = 0.239; *p* = 0.059) likely reflects the relative homogeneity of CIMT in this young cohort and the limited sample. Notably, the association between BMI category and family history of CVD (*p* = 0.040) suggests hereditary risk may potentiate weight-related vascular changes early in life, which is particularly relevant given the rising prevalence of overweight and obesity in Saudi Arabia [3].

Contrary to expectations, neither blood pressure (MAP group) nor ethnicity significantly influenced CIMT in this cohort. The absence of a MAP–CIMT association is consistent with the young age and predominantly normal blood pressure profile of participants, in whom the cumulative haemodynamic exposure necessary to produce measurable intima-media thickening has not yet been sufficient. This aligns with Stein et al. [7], who note that blood pressure’s predictive value for CIMT is most pronounced in older populations and those with established hypertension. The absence of an ethnicity effect may reflect the young age of the cohort, wherein environmental and epigenetic differences in vascular ageing have not yet manifested or may be attributable to the relatively small ethnic subgroup sizes.

The pattern of stronger associations for right CIMT compared with left CIMT is noted. While right–left asymmetry has been reported in some prior studies and may relate to haemodynamic or anatomical differences between sides [13,14], the present findings are based on a small pilot sample and may equally reflect measurement variability or statistical fluctuation. This observation should therefore be interpreted with considerable caution and should not be over-interpreted as a consistent biological finding. Future studies should measure CIMT bilaterally, report both sides with equivalent analyses, and formally test laterality effects in adequately powered samples.

The null findings for dietary habits and CIMT, despite a significant linear trend between increasing BMI and saturated fat consumption (*p* = 0.017), may reflect the limitations of self-reported dietary data subject to recall bias, the cross-sectional design precluding causal inference, and the young age of the cohort, wherein dietary effects on CIMT may not yet be detectable. These findings are consistent with the observation by Akbari-Sedigh et al. [17] that adherence to a healthy dietary pattern is associated with prevention of CIMT progression primarily in overweight and obese paediatric populations rather than in healthy young adults.

A key contribution of this study is the demonstration of the feasibility of the Butterfly iQ+ HHUD for CIMT measurement in a non-specialist research and educational setting. Prior work has supported POCUS feasibility for carotid assessment [18,19]. Our intra-operator repeatability data (ICC = 0.87 right, 0.85 left) extend these findings to CIMT measurement in young adults, supporting the feasibility of the Butterfly iQ+ HHUD in non-specialist settings. Formal inter-operator reliability and comparison with cart-based systems remain necessary before clinical deployment. The device’s portability and low cost position it as a promising tool for democratising cardiovascular risk assessment in resource-limited settings [20]. Equivalent exploratory stepwise regression models using left CIMT and mean CIMT as dependent variables confirmed that no predictor met the entry criterion for left CIMT, and sex showed only marginal significance for mean CIMT (*p* = 0.025), consistent with the weaker and non-significant anthropometric correlations observed for left CIMT in Table 3. These supplementary analyses support the robustness of right CIMT as the primary outcome for this cohort while highlighting the need for bilateral reporting in future studies.

### Limitations

This study has several limitations. First, the cross-sectional design precludes causal inference. Second, and critically, the final sample (n = 63) is substantially below the pre-specified power calculation target of n = 128; post hoc power analysis confirms achieved power of approximately 41% for medium effect sizes, classifying this as a pilot/feasibility study. All inferential findings, particularly non-significant results, should be interpreted with caution, given the elevated risk of Type II error. Third, the convenience sample drawn exclusively from medical students at a single private university in Riyadh does not represent the broader young adult population of Saudi Arabia and limits external validity; medical students are typically from higher socioeconomic backgrounds and may differ in health behaviours from the general population. Claims of establishing national reference data are therefore not supportable and have been reframed throughout as preliminary single-institution data. Fourth, the dietary questionnaire used in this study was not formally validated; it consisted of two self-report items adapted for this study and has not undergone psychometric testing. Reliance on this non-validated, self-reported dietary instrument is susceptible to recall and social desirability bias; future studies should employ validated food frequency questionnaires (e.g., the PREDIMED screener or Mediterranean Diet Score) or biomarker-based dietary assessment. Fifth, intra-operator ICC values for HHUD CIMT measurement were acceptable (ICC = 0.87 right, 0.85 left) but inter-operator reliability and direct comparison with a cart-based reference system remain to be established. Sixth, multiple statistical comparisons were performed without formal correction for multiplicity; borderline findings (e.g., BMI–CIMT ρ = 0.239; *p* = 0.059) should be interpreted exploratorily pending replication in adequately powered studies. Seventh, two participants with missing sex records were excluded listwise from sex-stratified analyses; although their exclusion is unlikely to alter the main findings, given the small number, this represents a minor source of potential bias that should be addressed through improved data collection protocols in future studies. Eighth, smoking status was not assessed; given the established association between tobacco use and increased CIMT, this represents a potential source of unmeasured confounding. Although smoking prevalence among medical students in Saudi Arabia is generally low, it cannot be assumed to be negligible, and future studies should incorporate a validated smoking assessment.

## 5. Conclusions

This pilot study provides preliminary CIMT reference data for young, healthy medical students at a single university in Riyadh, Saudi Arabia, using the Butterfly iQ+ HHUD. Given that the achieved study power was approximately 41%, these findings should be interpreted as hypothesis-generating and require confirmation in adequately powered, population-representative samples. The mean CIMT of 0.053 cm (0.53 mm) is consistent with the expected normal range for this age group and with Eastern Mediterranean regional reference values. Sex emerged as the sole independent predictor of right CIMT, with males demonstrating significantly greater carotid wall thickness than females, consistent with established biological sex differences in vascular physiology. Body weight and height showed significant positive correlations with right CIMT; while these associations are consistent with the broader literature, the modest effect sizes and pilot nature of the present study preclude definitive causal claims, and body composition should be regarded as a candidate early-life factor warranting further investigation in adequately powered prospective studies. Ethnicity, blood pressure, and dietary habits did not significantly influence CIMT in this young, asymptomatic cohort, though the limited sample size precludes definitive conclusions.

The Butterfly iQ+ HHUD demonstrated satisfactory feasibility as a point-of-care imaging tool for CIMT measurement in a non-specialist setting, with acceptable intra-operator repeatability (ICC = 0.87 right, 0.85 left). These findings provide a pilot methodological and empirical foundation for future large-scale, multi-site, population-representative longitudinal studies tracking the evolution of cardiovascular risk profiles in young Saudi adults. Future work should include formal inter-operator reliability testing and comparison with cart-based ultrasound systems. Integration of HHUD-based CIMT screening into preventive cardiovascular programmes at universities and community health centres in Saudi Arabia warrants consideration, contingent on further validation studies.

## Figures and Tables

**Table 1:** Descriptive statistics of the study sample (*n* = 63).

Variable	Mean	SD
Age (years)	19.19	1.89
Height (cm)	168.47	10.01
Weight (kg)	71.31	17.35
BMI (kg/m^2^)	24.93	4.72
Systolic BP (mmHg)	122.06	9.22
Diastolic BP (mmHg)	76.86	7.25
MAP (mmHg)	91.93	6.81
Right CIMT (cm)	0.0529	0.007
Left CIMT (cm)	0.0527	0.006
Mean CIMT (cm)	0.0529	0.006

SD, standard deviation; BMI, body mass index; BP, blood pressure; MAP, mean arterial pressure; CIMT, carotid intima-media thickness.

**Table 2:** Comparison of key variables between male and female participants.

Variable	Male (*n* = 30) Mean ± SD	Female (*n* = 31) Mean ± SD	*p*-Value
Height (cm)	176.28 ± 6.89	160.82 ± 6.14	<0.001 *
Weight (kg)	80.31 ± 17.15	63.40 ± 13.24	<0.001 *
BMI (kg/m^2^)	25.70 ± 4.56	24.49 ± 4.83	0.320
Systolic BP (mmHg)	123.63 ± 8.93	120.52 ± 9.13	0.183
Diastolic BP (mmHg)	77.27 ± 6.86	76.42 ± 7.90	0.657
MAP (mmHg)	92.72 ± 6.34	91.12 ± 7.31	0.364
Right CIMT (cm)	0.055 ± 0.0068	0.051 ± 0.0073	0.012 *
Left CIMT (cm)	0.054 ± 0.0056	0.052 ± 0.0070	0.300
Mean CIMT (cm)	0.0548 ± 0.006	0.0513 ± 0.007	0.031 *

* *p* < 0.05. SD, standard deviation; BMI, body mass index; BP, blood pressure; MAP, mean arterial pressure; CIMT, carotid intima-media thickness.

**Table 3:** Selected Spearman’s rho correlations with right CIMT.

Variable	ρ (Rho)	*p*-Value	Significance
Height (cm)	0.266	0.035	Significant *
Weight (kg)	0.320	0.011	Significant *
BMI (kg/m^2^)	0.239	0.059	NS
Age (years)	−0.140	0.275	NS
Systolic BP (mmHg)	0.083	0.515	NS
Diastolic BP (mmHg)	−0.070	0.585	NS
MAP (mmHg)	−0.020	0.877	NS

* Significant at *p* < 0.051. NS, not significant; BMI, body mass index; BP, blood pressure; MAP, mean arterial pressure.

## Data Availability

The data supporting the reported results are available from the corresponding author upon reasonable request, subject to ethical restrictions protecting participant confidentiality.

## Supplementary Materials

The following supporting information can be downloaded at: https://www.mdpi.com/article/10.3390/healthcare14121626/s1.
