# Supplementary material for: Carotid Intima-Media Thickness in Young Healthy Adults in Saudi Arabia: A Pilot Study of Preliminary CIMT Measurements and Cardiovascular Risk Assessment Using a Handheld Ultrasound Device
Source: Healthcare (Basel). 2026 Jun 9;14(12):1626. doi: 10.3390/healthcare14121626 (PMC13300029; doi:10.3390/healthcare14121626)
Supplement: Supplementary file 1 [file healthcare-14-01626-s001.zip › healthcare-4325567-supplementary.pdf]

Survey

Carotid Intima-Media Thickness in Young Healthy Adults in Saudi Arabia: A Pilot Study of Preliminary CIMT Measurements and Cardiovascular Risk Assessment Using a Handheld Ultrasound Device

The objective of this study is to evaluate the effectiveness and accuracy of handheld ultrasound devices in measuring the wall thickness of the carotid artery in young, healthy individuals in Saudi Arabia. This study is conducted by Dr. Shahmina and team. It has Alfaisal University IRB approval (No. IRB20317). The study aims to determine whether these portable devices can be used as a reliable tool to identify individuals at risk of developing cardiovascular diseases by assessing carotid artery wall thickness. Additionally, the study seeks to explore the correlation between various cardiovascular risk factors and carotid artery wall thickness in this demographic.
Your participation is entirely voluntary. You may withdraw at any time without penalty and that withdrawal will not affect their eligibility for compensation, grades, evaluations, academic standing, access to resources, or professional opportunities including communication with faculty, inclusion in any course-related opportunities, recommendations, or any other aspect of their current or future education or employment opportunities.

Inclusion criteria for recruiting asymptomatic adults without evidence of clinical atherosclerosis. included the following:
1. Age of 18 years or older male and female.
2. Ability to understand and give written consent.
3. Tolerant of carotid artery sonography

The exclusion criteria any history of transient ischemic attack, neuromuscular disorder, or significant diseases of the liver, kidney and heart diagnosed at the time of ultrasound examination.
Do you consent to participate in the study?   (Yes, No)
If yes, then the following information will be collected.

1. Age - Years: ________________________

2. Nationality:
   - 1. Saudi
   - 2. Non-Saudi (specify) ____________

3. Gender:
   - 1. Male
   - 2. Female

4. Do you have any cardiovascular illness?
   - 1. No
   - 2. Yes (specify) ____________

5. Do you have a family history of any cardiovascular disease?
- 1. No
- 2. Yes (specify) _____________

6. Dietary habits:

6.1 - How often do you consume fruits and vegetables in your diet?

1. Rarely or never
2. Occasionally (1-2 servings per day)
3. Moderately (3-4 servings per day)
4. Regularly (5 or more servings per day)

6.2 - How often do you consume foods high in saturated fats, such as red meat, fried foods, or processed snacks?

1. Rarely or never
2. Occasionally (1-2 times per week)
3. Moderately (3-4 times per week)
4. Regularly (5 or more times per week)

7. Height in cm: _________________________

8. Weight in kg: _________________________

9. Blood pressure in mmHg: ____________/____________

10. Carotid artery wall thickness measured by ultrasound (US) in cm: Right side (-------), Left side (--------)

Please fill out the survey completely and accurately. Thank you for your participation in our study! In case of need please contact the undersigned principal investigator.

Dr Shahmina Naz,
Principal Investigators
Department of Anatomy and Genetics,
COM, Alfaisal University.
snaz@alfaisal.edu
Mobile: 0542596705
